# Epidemiology of Pediatric Essential Tremor in the United States: A Systematic Literature Review from 2010 to 2020

**DOI:** 10.5334/tohm.681

**Published:** 2022-04-19

**Authors:** Margaret E. Gerbasi, Adi Eldar-Lissai, Kelly E. Lyons

**Affiliations:** 1 Sage Therapeutics, Inc., Cambridge, MA, USA; 2 University of Kansas Medical Center, Kansas City, KS, USA

**Keywords:** essential tremor, epidemiology, pediatric, children, age of onset

## Abstract

**Background:**

Essential tremor (ET) is one of the most common movement disorders worldwide, yet the size of the pediatric ET population is not well understood. The objective of this review was to identify, evaluate, and synthesize evidence describing the epidemiology of pediatric ET in the United States published between 2010 and 2020.

**Methods:**

The authors searched MEDLINE, Embase, and the Cochrane Database of Systematic Reviews using terms related to ET, epidemiology, and pediatric patients. Eligibility criteria included observational studies that reported primary data on pediatric prevalence or incidence of ET or age of onset/diagnosis of ET. A total of 562 unique articles were identified for screening.

**Results:**

The review did not identify any studies that reported information on pediatric prevalence or incidence of ET, or age of ET diagnosis among nonpediatric patients. A total of 10 samples were identified, all of which described age of ET onset that ranged from 27.0 years to 56.7 years among 9 adult populations (weighted mean of 41.6 years) and 9.7 years in a single pediatric sample. One adult sample reported that 13% of all ET cases reported onset by age 14, and 21.8% of all ET cases reported onset by age 18.

**Discussion:**

There is a notable lack of recent data describing the incidence and prevalence of pediatric ET in the United States. Many children who present with symptoms of ET may not be diagnosed until later in life, and an increased awareness of pediatric ET could allow for early identification and monitoring of these patients.

## Introduction

Essential tremor (ET) is defined as an isolated tremor syndrome of bilateral upper limb action, sometimes accompanied by tremor in other locations, that occurs in the absence of other neurological signs such as ataxia and parkinsonism [1]. Some affected patients may experience additional neurologic symptoms, including memory impairment and difficulties with gait or posturing [2,3]. For many patients, ET leads to significant impairment in the ability to perform activities of daily living and loss of independence [4,5]. In addition to physical symptoms, patients with ET often suffer from psychiatric symptoms such as worrying, fatigue, and embarrassment [6,7]. These patients have also demonstrated higher rates of psychiatric comorbidities, such as depression, anxiety, and sleep disturbances, compared with healthy controls [8].

ET is one of the most common movement disorders worldwide and has been reported to occur in approximately 1% of the global population [9,10,11]. Among people aged 65 years and older, this prevalence increases to nearly 6% [9]. In the United States, the prevalence of ET among adults was estimated to be 2.6% in 2018 (6.4 million people), which included an estimated rate of 8.2% in people aged 85 years and older [12]. Although ET has often been characterized as a disease that predominantly affects the elderly, some studies have shown a bimodal distribution in the age of onset that peaks during the second and sixth decades of life [13,14]. ET is familial in approximately half of patients (though estimates vary widely based on the definition used; for example, the presence of at least one first- or second-degree relative with any tremor provides a more liberal estimate, whereas a more conservative estimate is obtained when a specific diagnosis of ET is required among the same relatives) [2,3,15], and some studies have suggested that patients with childhood-onset ET are more likely to have familial ET [15,16,17].

The size of the pediatric ET population is not well understood. A literature review published in 2009 was unable to identify published epidemiological survey data from any country that focused on ET exclusively in children, and found only 5 studies that reported prevalence data in age-based subgroups that included children [18]. Based on this identified paucity of data prior to 2010, the objective of this review was to identify, evaluate, and synthesize evidence describing the incidence and prevalence of pediatric ET, age of ET onset, and age of ET diagnosis, based on published data in the United States between 2010 and 2020.

## Methods

A systematic literature search for studies that examined the epidemiology of pediatric ET was conducted with methods consistent with the Preferred Reporting Items for Systematic Reviews and Meta-Analyses (PRISMA) reporting guidelines [19]. Databases searched included Embase (via *Embase.com*), MEDLINE (via PubMed), and the Cochrane Database of Systematic Reviews (via the Cochrane Library); search terms included those related to ET, epidemiology, and pediatric populations (including infants, children, and adolescents). Searches were conducted in February 2020 and were restricted to the last 10 years for database searches and the previous 5 years for conference proceedings (see Appendix Tables S1-3 for detailed search strategies).

Studies were included if they reported primary data on pediatric prevalence or incidence of ET or age of onset/diagnosis of ET. In addition, studies were required to include a US-based sample, be observational in design, and be published in English (see Appendix Table S4 for detailed inclusion and exclusion criteria). Search results were screened by 2 reviewers initially by titles and abstracts, followed by a review of the full text by a single reviewer with a second reviewer screening a random sample of 10% of studies. Any disputes were resolved through discussion between reviewers or consultation with a third reviewer.

Data from included studies were extracted by a single reviewer with accuracy confirmed by a second reviewer. The extracted data described the study methodology, patient demographic and clinical characteristics, and ET prevalence, incidence, and age of onset/diagnosis. The quality of included studies was assessed by a single reviewer using a modified version of the Newcastle-Ottawa scale for observational studies that focused exclusively on the selection rating (maximum score of 5) and outcome/exposure rating (maximum score of 3) [20,21]; comparability ratings were not assessed based on lack of comparative groups in most included studies.

Age of ET onset was summarized using a pooled data analysis, which was stratified for key sample characteristics such as the location of the sample (clinical or community) and the classification of ET (familial or sporadic). Initially, each participant was weighted equally (referred to below as the weighted mean age of onset), and an additional analysis was conducted in which familial and sporadic distributions of age of onset were evaluated and combined in equal weights.

## Results

**Figure 1:**
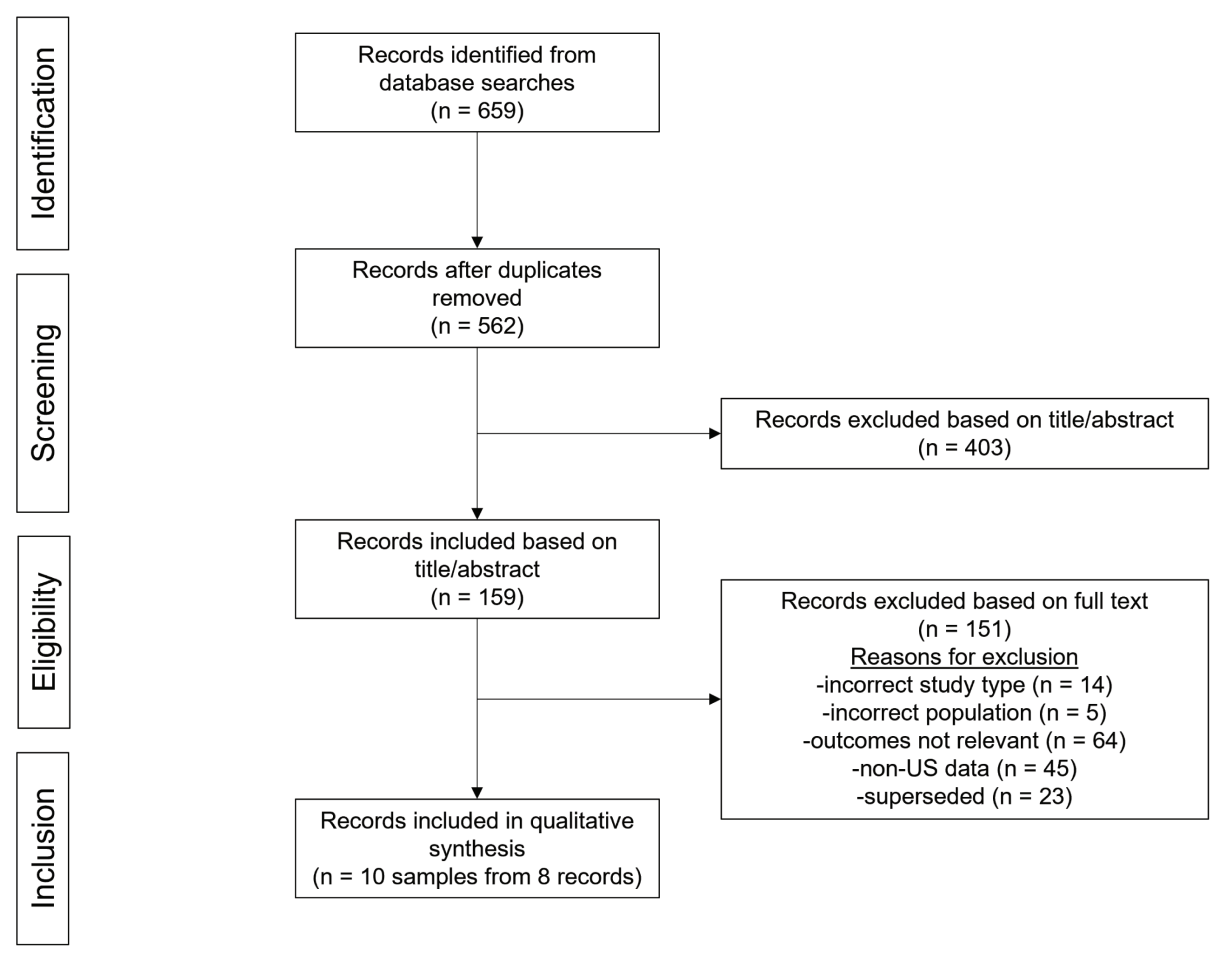
**PRISMA diagram of study selection.** PRISMA, Preferred Reporting Items for Systematic Reviews and Meta-Analyses.

A total of 562 unique articles were identified for screening; of these, 159 were selected for screening by full text. Overall, 8 articles representing 10 independent samples met the inclusion criteria (***Figure 1***); these included 1 pediatric-only sample and 9 samples from broader populations (***Table 1***) [15,22,23,24,25,26,27,28]. Four samples were specific to familial ET [22,23,25], and another study reported data for familial ET as a subgroup [15]. Nearly all samples were defined as “clinical” samples because patients were diagnosed, i.e., “definite clinical” samples (or presumed to be diagnosed based on limited information provided, i.e., “probable clinical” samples), in a clinical setting, whereas one study included a sample that recruited patients from a community setting [23].

**Table 1:** Study characteristics and age of ET onset for included samples.


AUTHOR, YEAR	SAMPLE POPULATION	DESIGN	DIAGNOSTIC CRITERIA	PATIENT CHARACTERISTICS	AGE OF ONSET

*SAMPLES INCLUDING ADULTS*					

Hedera 2013 [22]	Vanderbilt/University of Tennessee (probable clinical); familial ET only; N = 104	Cross-sectional	ET: presence of bilateral postural and kinetic tremors Familial: ≥2 meioses from a proband	Mean age: 58 ± 7.5 years Mean WHIGET score: 14.8 ± 4.6 Female: 61.5% Familial: 100%	Mean age: 32.1 ± 11.4 years Range: 8–50

Louis 2015a [15]^a^	CUMC 2000–2009 (clinical); N = 376	Cross-sectional	ET: WHIGET criteria Familial: self-report (≥1 relatives with ET or tremor)	Mean age: 67.5 ± 15.1 years Mean WHIGET score: 18.8 ± 7.2 Female: 52.1% Familial ET: 62%	Mean age: 44.7 ± 22.5 years

Louis 2015b [23]^b^	ETCBR 2003–2014 (clinical); N = 177	Prospective cohort	ET: WHIGET criteria Familial: self-report of first- or second-degree relatives	Mean age: 83.7 ± 5.8 years Mean WHIGET score: 24.2 ± 6.7 Female: 61.0% Familial: 27.1%	Mean age: 42.4 ± 22.9 years

	WHIGET 1995–2000 (community); familial ET only; N = 106	Prospective cohort	ET: WHIGET criteria Familial: self-report of first- and second-degree relatives	Mean age: 69.8 ± 18.4 years Mean WHIGET score: 16.4 ± 6.7 Female: 59.4% Familial: 100%	Mean age: 56.7 ± 25.6 years

	FASET phase 1 2011–2014 (clinical); familial ET only; age of onset ≤50 years; N = 160	Cross-sectional	ET: physician diagnosis, confirmed by WHIGET criteria Familial: ≥2 living relatives with physician diagnosis of ET	Mean age: 60.0 ± 18.0 years Mean WHIGET score: 20.1 ± 5.2 Female: 51.3% Familial: 100%	Mean age: 27.0 ± 17.8 years

Louis 2018 [25]	FASET phase 2 2015–2018 (clinical); other criteria as in phase 1; N = 203 (98 probands; 105 affected relatives)	Cross-sectional	As in phase 1	Mean age, years Probands: 68.8 ± 11.2 Affected relatives: 60.9 ± 16.2 Mean WHIGET score Probands: 23.5 ± 6.5 Affected relatives: 17.7 ± 5.9 Female Probands: 59.2% Affected relatives: 60.0% Familial: 100%	Mean age, years Probands: 28.4 ± 19.4 Affected relatives: 33.4 ± 19.9

Louis 2016 [24]	COGNET 2014–2015 (clinical); age ≥55 years; N = 100	Prospective cohort	ET: physician diagnosis, confirmed by WHIGET criteria Familial: self-report	Mean age: 80.5 ± 8.1 years Mean WHIGET score: 21.5 ± 5.8 Female: 55.0% Familial: 50.0%	Mean age: 39.1 ± 21.2 years Range: 1–83

Ortega-Cubero 2015 [26]	Mayo Clinic (probable clinical); N = 257 cases; 697 controls	Case control	ET: diagnosis by movement disorder specialist Familial: NR^c^	Mean age: 73.42 ± 11.73 years Mean WHIGET score: NR Female: 53.3% Familial: NR	Mean age: 50.66 ± 19.98 years Range: 5–88

Ross 2011 [27]	Emory University (probable clinical); N = 118 cases; 268 controls	Case control	ET: diagnosis by movement disorder specialist Familial: NR	Mean age: 69.6 ± 12.3 years Mean WHIGET score: NR Female: 57% Familial: NR	Mean age: 46.0 ± 21.7 years Range: 1-83

***PEDIATRIC-ONLY SAMPLES***					

Ghosh 2017 [28]	Cleveland Clinic 1984–2011 (clinical); age <21 and ET onset <18 years; N = 211	Retrospective cohort	ET: bilateral action tremor of the hands and forearms Familial: based on chart review	Mean age: 14.09 ± 5.00 years^d^ Mean WHIGET score: NR^e^ Female: 38% Familial: 35%	Mean age: 9.71 ± 5.62 years


Data are presented as mean ± standard deviation unless otherwise indicated. ^a^ Additional study design data acquired from Louis et al., 2013 [45]. ^b^ Additional study design data acquired from Louis et al., 2005 [46] (ETCBR); Louis et al., 1997 [29] (WHIGET); Louis et al., 2013 [47] (FASET). ^c^ Only 1 family member per patient included. ^d^ Mean age at presentation. ^e^ 55% reported functional impairment; 29.4% required medication. COGNET, Clinical Pathological Study of Cognitive Impairment in Essential Tremor; CUMC, Columbia University Medical Center; ET, essential tremor; ETCBR, Essential Tremor Centralized Brain Repository; FASET, Family Study of Essential Tremor; NR, not reported; WHIGET, Washington Heights-Inwood Genetic Study of Essential Tremor Rating Scale.

No studies reported information on pediatric prevalence or incidence of ET, or age of diagnosis of ET.

### Age of onset

Of the 9 broader population (non-pediatric) ET samples, mean ages of patients at the time of assessment ranged from 58 to 84 years, and female patients comprised between 51% and 62% of the sample groups (***Table 1***). The proportion of patients with familial ET among studies that were not restricted to familial-only (4 samples) ranged from 27% to 62%.

Tremor severity was rated using the Washington Heights-Inwood Genetic Study of ET Tremor Rating Scale (WHIGET) total score in 7 samples. This scale is based on a 23-item examination in which patients are assessed in various postures and when undertaking different movements, with a tremor rating between 0 (no tremor) and 3 (large amplitude tremor) assigned to each item [29]. Mean WHIGET total scores ranged from 14.8 to 24.2 across the identified samples (***Table 1***).

Mean age of ET onset among samples that included adults ranged from 27.0 years to 56.7 years, although this was impacted by age-based inclusion criteria in some samples (***Table 1***). In a pooled analysis of all samples that included adults (N = 1601), the weighted mean age of onset was 41.6 years [15,22,23,24,25,26,27]. Age of onset was impacted by whether patients were identified in the clinic or community. When limited to clinical samples (both definite and probable samples; N = 1495), weighted mean age of onset decreased to 40.5 years [15,22,24,25,26,27]. When further restricted to definite clinical samples (N = 1016), this decreased further to 38.2 years [15,24,25]. In the single community sample identified by the review (N = 106), weighted mean age of onset was 56.7 years [23]. In patients with familial ET (N = 805), the weighted mean age of onset was 36.1 years [15,22,23,25].

**Figure 2:**
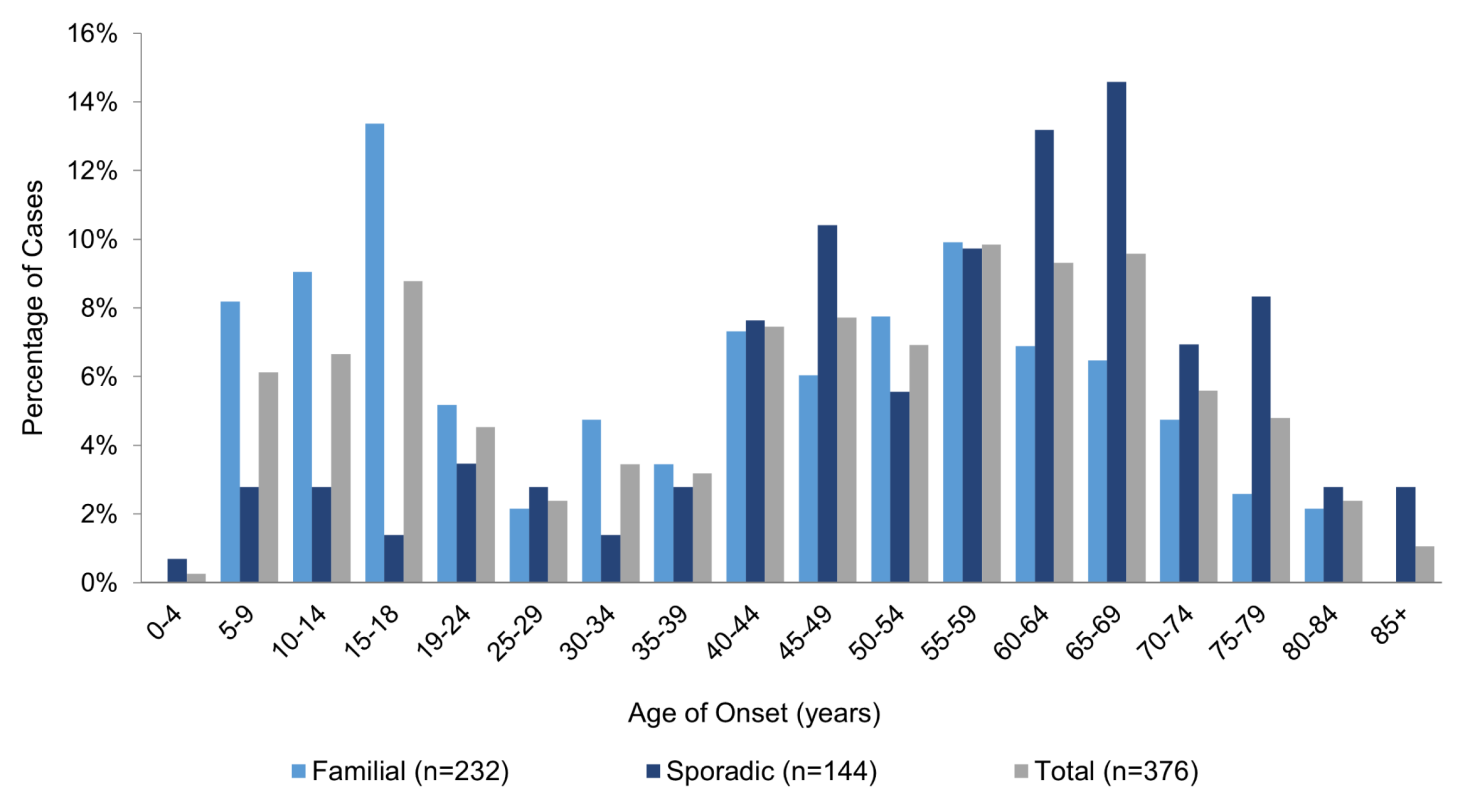
**Age of onset distribution for patients with ET from the Columbia University Medical Center.** Adapted from Louis et al., 2015 [15]. ET, essential tremor.

Additional information on the age of ET onset was available for one sample (N = 376) from the Columbia University Medical Center [15]. This study provided evidence supporting earlier studies showing that the age of onset distribution appears to be bimodal, with peaks in the second and sixth-to-seventh decades of life, and also demonstrated that the early peak may be predominantly driven by familial cases (***Figure 2***) [15]. In total, 13% of all ET cases (17.2% familial/6.3% sporadic) reported onset by age 14, and 21.8% of all ET cases (30.6% familial/7.6% sporadic) reported onset by age 18 [15]. When the sample was reweighted to a 50% familial distribution, 11.7% and 19.1% of patients were estimated to have ET onset by ages 14 and 18, respectively.

In the single retrospective chart review study that was restricted to pediatric patients only (N = 211), mean age of onset was 9.7 years and mean age at presentation was 14.1 years [28]. Twenty-eight patients (13%) indicated that they were uncertain about timing of onset, whereas 29% reported onset within the first decade of life and 57% in the second decade [28]. Most patients (71%) did not require medication for ET, and no ET-related functional impairment was observed from the charts of 45% of all patients. None of the 211 pediatric patients had disability so severe that it impaired daily activities [28].

### Quality assessment

Most studies were assessed to be somewhat representative of the average ET population in each community or clinic based on the Newcastle-Ottawa criteria, as the studies had relatively broad inclusion criteria and used known diagnostic criteria, such as WHIGET. Although some studies did restrict the age of included patients, this is less likely to impact the age of onset findings, particularly among older population samples. No studies reported a high-quality outcome assessment method such as record linkage or independent blind assessment; instead, many relied on self-report, while the lowest scoring studies lacked a clear description of the method of outcome assessment. Complete quality assessment scores can be found in the Appendix (Table S5).

## Discussion

No studies published from 2010 to 2020 were identified that reported incidence or prevalence data for pediatric ET, or information regarding the age of diagnosis, within the United States. A previously published US population-based analysis (using data collected between 1935 and 1979 in Rochester, Minnesota) reported an age- and sex-adjusted incidence rate of ET of 23.7 per 100,000 (1965–79) and a prevalence of 305.6 per 100,000 in 1979 [30]. Among patients aged 0-19 years in this sample, the incidence rate was much lower by comparison at 2.9 per 100,000 over the same period [30]. Studies from other countries have reported prevalence rates of pediatric ET that were very low or effectively zero in community samples [31,32,33].

Mean age of onset findings from our review ranged between mid-20s and mid-50s (pooled analysis of weighted mean age: 42 years) and support the presence of a bimodal pattern. However, it should be noted that mean age of onset is a limited measure for describing such bimodal patterns, and additional studies that report the distribution of onset ages would be useful to further elucidate the epidemiology of ET among the pediatric population. Similar bimodal patterns of ET onset age have been demonstrated in other countries [34,35].

There were notable differences in mean ages of onset, with earlier onset observed in clinical samples compared with the single community sample, and earlier onset observed for patients with familial ET only compared with broader ET populations. These findings could suggest that patients who are identified at an earlier age may be those with the most pronounced/severe disease (making them more likely to be identified from clinical samples versus community samples), whereas community samples may be more likely to capture a broader proportion of older patients who have mild ET. It is possible that milder pediatric cases may be more frequently unidentified or misdiagnosed, while mild ET may be more commonly considered in the differential diagnosis in older patients [36].

Findings also suggest that genetic predisposition to ET could lead to an earlier onset of disease, which has been similarly demonstrated by analyses beyond the scope of this review [16]. Other studies have shown potential differences in the phenotype of ET between patients with early and late onset. In one study, those with onset by age 22 years were more likely to have lower limb tremor and less likely to have head and voice tremor compared with those who had ET onset at age 36 years or later [37]. In another study, patients with ET onset up to age 30 were more likely to have familial ET and lower limb tremor, as well as a significantly longer duration of tremor symptoms, compared with patients with ET onset occurring at ages 55 years and older [35].

Collectively, these findings may suggest that many children who present with symptoms of ET remain undiagnosed until later in life [36]. Some reasons for this could include the fact that children may hide their symptoms; parents, teachers, and other caregivers may not have an adequate understanding of childhood-onset ET to recognize the symptoms; and healthcare providers may misdiagnose ET as a different disorder. There are limited data in the literature that assess potential reasons for late diagnosis, indicating a possible need for increased awareness of pediatric ET that should be supported by additional research. The single pediatric population sample identified by this review showed that there was a 4–5-year gap between mean ages of disease onset and presentation, and also noted that children with ET demonstrated less functional impairment compared with what is typically observed in adults [28]. This is supported by an earlier study in which only 24 of 39 patients with pediatric ET from a clinical sample were receiving active treatment for tremors [36], suggesting that the disease was relatively mild for many of these patients.

Another considerable challenge arises because the difference between physiologic tremor and ET has been well characterized in adults but not children. In a study of 819 children in Spain, mild but consistent tremor was observed in at least one hand for 52% of participants and, although the authors concluded that this was likely due to physiologic tremor rather than ET, the proportion of patients with either underpinning cause remained unclear [38]. In addition, the currently established assessments for ET do not appear to have been validated in pediatric samples. Even among adults, several sets of criteria with different degrees of stringency have been proposed over time to identify patients with ET [1,39,40]. For instance, the International Parkinson and Movement Disorder Society’s consensus definition of ET requires at least 3 years of symptom duration, potentially limiting its applicability in children [1]. Furthermore, the accuracy of assessments for other movement disorders such as ataxia and dystonia improve considerably as children mature into early adolescence [41,42,43], and a study of hand tremor in 287 children found a distinct difference in tremor frequency pattern between those aged 2 to 9 years compared with those aged 10 to 16 years (higher frequency in the older cohort) [44]. If the same age-related impact on tremor and other symptoms were to hold true for children with ET it could necessitate further study and revisions to the diagnostic criteria to account for both normal child neurologic development and the likelihood that children will experience milder ET compared with adults when it first presents.

### Limitations

Limitations of this review include restrictions to both the period of the literature searched and the geographic location of included studies. In particular, only studies published between 2010 and 2020 were included, although this limit was based on the presence of a comprehensive literature review of global epidemiology studies published in 2009 [18]. In this review, only US-based samples were considered, therefore potentially relevant pediatric ET data from other countries were not identified. In addition, the large proportion of clinical samples in our review (9 out of 10 total samples, including 8 samples in adults) may result in under-representation of age of onset and other epidemiology data in a community-based population. Finally, the studies identified by this review are difficult to compare directly as they do not use consistent criteria for defining ET and in some cases do not account for neurologic comorbidities that could impact the presentation of ET-related symptoms.

### Conclusions

Overall, this systematic review revealed a considerable data gap for US-based pediatric ET epidemiology in the literature. Although there are data suggesting that many patients retrospectively recall the onset of ET by age 18, there remains limited information to support the true epidemiology of pediatric ET, including the rate at which patients are diagnosed or treated. Future research to further elucidate the epidemiology of pediatric ET should first aim to clarify whether ET diagnostic criteria require adaptation when applied to children, followed ideally by long-term prospective cohort studies during which regular assessments are made to identify cases of ET throughout childhood development. Though it may be the case that many patients present with less severe disease in childhood, this could represent an opportunity for early identification and monitoring to ensure that adequate treatment and supportive care is available to patients when required.

## Additional File

The additional file for this article can be found as follows:

10.5334/tohm.681.s1 Supplementary Appendix. Tables S1 and S5.
