# Supplementary material for: Epidemiology of Pediatric Essential Tremor in the United States: A Systematic Literature Review from 2010 to 2020
Source: Tremor Other Hyperkinet Mov (N Y). 2022 Apr 19;12:11. doi: 10.5334/tohm.681 (PMC9029662; doi:10.5334/tohm.681)
Supplement: Supplementary Appendix. — Tables S1 and S5. [file tohm-12-1-681-s1.pdf]

Supplementary Appendix

Table S1. MEDLINE search strategy

No.	Category	Search String
**1**	Disease terms	("essential tremor"[Mesh] OR ("essential tremor"[Title/Abstract] OR "kinetic tremor"[Title/Abstract] OR "benign essential tremor"[Title/Abstract] OR "familial tremor"[Title/Abstract] OR "hereditary essential tremor"[Title/Abstract]))
**2**	Outcome terms	("prevalence"[tw] OR "prevalent"[tw] OR "incidence"[tw] OR "incident"[tw] OR "epidemiology"[tw] OR "epidemiologic"[tw] OR "population"[tw]) <br>OR ("infant"[MeSH Terms] OR "child"[MeSH Terms] OR "children"[MeSH Terms] OR "adolescent"[MeSH Terms] OR "juvenile"[MeSH Terms]) <br>OR <br>("infant"[tw] OR "child"[tw] OR "children"[tw] OR "adolescent"[tw] OR "juvenile"[tw] or “pediatric”[tw])
**3**	Combination	**#1 AND #2**
**4**	Publication type limits	("Comment"[pt] OR "Editorial"[pt] OR "Letter"[pt] OR "News"[pt] OR "Published Erratum"[pt] OR "Retraction of Publication"[pt] OR "Case Reports"[pt] OR "Biography"[pt] OR "Expression of Concern"[pt] OR "Patient Education Handout"[pt] OR "Directory"[pt] OR "Newspaper Article"[pt] OR "Historical Article"[pt] OR "Retracted Publication"[pt] OR commentary[ti] OR editorial[ti] OR "letter to editor"[ti] OR erratum[ti] OR correction[ti] OR congresses as topic [mesh] OR (animals[mh] not (animals[mh] and humans[mh]))
**5**	Combination	**#3 NOT #4**
**6**	**Filters**: Abstract, English,	**#5 AND** ("english"[Language]) **AND** "hasabstract"[All Fields]
**7**	Date range: 2010 to Feb 01, 2020	**#6 AND** ("2010"[Date - Publication] : "2020/02/01"[Date - Publication])

Table S2. Embase search strategy

No.	Category	Search String
**1**	Disease terms	'essential tremor'/exp OR 'essential tremor':ti,ab OR 'kinetic tremor':ti,ab OR 'benign essential tremor':ti,ab OR 'familial tremor':ti,ab OR 'hereditary essential tremor':ti,ab
**2**	Outcome terms	(‘prevalence’:ti,ab OR ‘prevalent’:ti,ab OR ‘incidence’:ti,ab OR ‘incident’:ti,ab OR ‘epidemiology’:ti,ab OR ‘epidemiologic’:ti,ab OR ‘population’:ti,ab OR ‘infant’:ti,ab OR ‘child’:ti,ab OR ‘children’:ti,ab OR ‘adolescent’:ti,ab OR ‘juvenile’:ti,ab OR ‘pediatric’:ti,ab)
**3**	Combination	**#1 AND #2**

No.	Category	Search String
**4**	Publication type limits	'editorial'/exp OR 'book'/exp OR 'erratum'/exp OR 'letter'/exp OR 'note'/exp OR 'conference paper'/exp OR 'in vitro study'/exp OR 'in vivo study'/exp OR 'cell culture'/exp OR 'cell line'/exp OR 'mathematical model'/exp OR 'theoretical model'/exp OR 'feasibility study'/exp OR 'pilot study'/exp OR [editorial]/lim OR [erratum]/lim OR [letter]/lim OR [note]/lim OR ('animal'/exp NOT ('animal'/exp AND 'human'/exp))
**5**	Combination	**#3 NOT #4**
**6**	**Filters**: Abstract, English	**#5 AND** [english]/lim AND [abstracts]/lim
**7**	Date range: 2010 to Feb 01, 2020	#6 AND [1-1-2010]/sd NOT [2-2-2020]/sd AND [2010-2020]/py
**8**	Conference abstracts limited to last five years	**#7 AND** ([conference abstract]/lim OR [conference paper]/lim OR [conference review]/lim) AND [2010-2014]/py
**9**	All limits	**#7 NOT #8**

Table S3. Cochrane search strategy

No.	Category	Search String
**1**	Disease terms	MeSH descriptor: [Essential Tremor] explode all trees OR ("essential tremor" OR "kinetic tremor" OR "benign essential tremor" OR "familial tremor" OR "hereditary essential tremor"):ti,ab,kw
**2**	Outcome terms	(“prevalence” OR “prevalent” OR “incidence” OR “incident” OR “epidemiology” OR “epidemiologic” OR “population” OR “infant" OR “child” OR "children” OR “adolescent” OR “juvenile” OR “pediatric”)
**3**	Combination	**#1 AND #2**
**4**	Date range: 2010 to Feb 01, 2020	with Cochrane Library publication date from Jan 2010 to Feb 2020
**5**	Publication type terms	In Cochrane reviews

Table S4. Inclusion and exclusion criteria

Parameter	Inclusion Criteria	Exclusion Criteria
Population	Pediatric patients diagnosed with ET, including familial ET<br>- Any disease severity (mild, moderate, or severe)<br>- Retrospective reporting of pediatric ET onset or diagnosis will be considered for estimation of the pediatric ET population but will be flagged accordingly	• Studies limited to adult ET patients that do not report pediatric incidence or prevalence, age of onset, or age of diagnosis
Intervention/ Comparator	NA	NA

Parameter	Inclusion Criteria	Exclusion Criteria
Outcomes	• Epidemiology of pediatric ET, including incidence and prevalence<br>• Age of ET onset<br>• Age of ET at diagnosis	Studies that do not report pediatric incidence or prevalence, onset, or diagnosis
Study Design	• Observational studies – claims analysis, registry studies, surveys, electronic medical chart review, case-control studies, cohort studies<br>• Relevant SLRs and non-systematic reviews (for bibliographic check and search validation)	Preclinical studies, genetic models, animal studies, case reports, case studies
Geographical Limits	• No limit for search, US for extraction	NA
Publication Type Limits	• Peer-reviewed literature<br>• Conference abstracts published in the last 5 years<br>• English language	Conference abstracts older than 5 years, editorials, commentary, letter to editor, errata, expert opinions, narrative reviews
Temporal Limits	January 1, 2010 to February 1, 2020	NA

ET, essential tremor; NA, not applicable; SLR, systematic literature review.

Table S5. Newcastle-Ottawa quality assessment scores

Author, year	Selection Rating					Outcome Rating		
	(1)	(2)	(3)	(4)	Total	(1)	(2)	Total
Ghosh 2017 (1)	*	-	-	**	3	*	*	2
Hedera 2013 (2)	-	-	-	**	2	-	*	1
Louis 2015a (3)	*	-	-	**	3	*	*	2
Louis 2015b (4)								
ETCBR	*	-	-	**	3	*	*	2
WHIGET	*	-	-	**	3	*	*	2
FASET1	*	-	-	**	3	*	*	2
Louis 2016 (5)	*	-	-	**	3	*	*	2
Louis 2018 (6)	*	-	-	**	3	*	*	2
Ortega-Cubero 2015 (7)	-	*	-	**	3	-	*	1
Ross 2011 (8)	-	*	-	**	3	-	*	1

Selection questions include: (1) Representativeness of the sample; (2) Sample size; (3) Non-respondents; (4) Ascertainment of the exposure.

Outcome questions include: (1) Assessment of outcome; (2) Statistical test.

Assessments were made using criteria based on cross-sectional study design. Although not all studies were cross-sectional, age of onset was the only outcome reported and was consistently assessed in a cross-sectional manner.

References

1. Ghosh D, Brar H, Lhamu U, Rothner AD, Erenberg G. A series of 211 children with probable essential tremor. Mov Disord Clin Pract. 2017;4(2):231-6.
2. Hedera P, Davis TL, Phibbs FT, Charles PD, LeDoux MS. FUS in familial essential tremor - the search for common causes is still on. Parkinsonism Relat Disord. 2013;19(9):818-20.
3. Louis ED, Clark LN, Ottman R. Familial versus sporadic essential tremor: what patterns can one decipher in age of onset? Neuroepidemiology. 2015;44(3):166-72.
4. Louis ED, Hernandez N, Michalec M. Prevalence and correlates of rest tremor in essential tremor: cross-sectional survey of 831 patients across four distinct cohorts. Eur J Neurol. 2015;22(6):927-32.
5. Louis ED, Collins K, Rohl B, Morgan S, Robakis D, Huey ED, et al. Self-reported physical activity in essential tremor: Relationship with tremor, balance, and cognitive function. J Neurol Sci. 2016;366:240-5.
6. Louis ED, Hernandez N, Sebastian AA, Clark LN, Ottman R. Validity of probands' reports and self-reports of essential tremor: data from a large family study in North America. J Neurol Sci. 2018;393:45-50.
7. Ortega-Cubero S, Lorenzo-Betancor O, Lorenzo E, Agúndez JA, Jiménez-Jiménez FJ, Ross OA, et al. TREM2 R47H variant and risk of essential tremor: a cross-sectional international multicenter study. Parkinsonism Relat Disord. 2015;21(3):306-9.
8. Ross OA, Conneely KN, Wang T, Vilarino-Guell C, Soto-Ortolaza AI, Rajput A, et al. Genetic variants of α-synuclein are not associated with essential tremor. Mov Disord. 2011;26(14):2552-6.
